# Round the Hospitals

**Published:** 1916-10-21

**Authors:** 


### THE MATRONS' AND SISTERS' DEPARTMENT.

### ROUND THE HOSPITALS.

The announcement of the constitution and *personnel* of the Scottish Board of the College of Nursing has been but little noticed in the Scottish newspapers. The Board, however, is considered to be a good working one to start with. The constitution was drafted by the Scottish Conference, which has been sitting during the summer, and was accepted by the College Council. The question of the representation of the smaller hospitals has been left for adjustment later; and the provision for the co-optation of seven nurses should allow for the representation of all interests. In addition to the names we published last week the following have now consented to serve:—Miss Edmondson, matron, Royal Infirmary, Aberdeen; Dr. Ebenezer Duncan, President of the Royal Faculty of Physicians and Surgeons of Glasgow; Professor Ritchie, convener of house committee, Royal Infirmary, Edinburgh.

A new institution for the control of the profession of massage and the examination of candidates, to be called the Institute of Massage and Remedial Gymnastics, is in course of formation at Manchester. For some time past, so states the honorary secretary, it has been felt that it would be greatly to the advantage of the public, the medical profession, and massage generally if a widely representative Society were established which would be directly under medical control, and have a board of management elected annually upon which charitable institutions and the medical profession would be represented. The new Society has been founded with this object. Its executive committee are now engaged in the selection of a Council to be submitted for nomination to the first general meeting to be held in Manchester on November 10, and hospitals are being asked to nominate a representative to sit upon the Council of the new body for the coming year. The Institute has been incorporated with limited liability, with the licence of the Board of Trade to dispense with the word "Limited." Its honorary secretary is Dr. A. E. Barclay, M.D., and the eleven founders, who are all Manchester residents, include the chairman, general superintendent, and matron of the Royal Infirmary, and two members of its honorary staff, the chairman of Ancoats Hospital, the matron of Salford Royal Hospital, and the principal of a massage school. The temporary office is 40 Brazennose Street, Manchester. The Institute claims to be the only one of its kind admitting men to membership.

Many complaints and much criticism have reached us in regard to the hours fixed in numerous hospitals and kindred institutions for the lectures to probationer nurses in training. It is declared that in a number of these institutions, where such lectures and classes are held in the evening, the probationers are often so tired as to be incapable of giving any real attention to the lecture, nor have they adequate energy to enable them to attend closely to class work or to make the necessary notes for their future guidance. This ought not to be. Competent nurses cannot be created at training schools where the evils complained of exist.

The duty of arranging the annual staff holidays has this year been no easy one for many matrons. Miss Byles, the matron of Lambeth Infirmary, has had a particularly difficult task. Usually holidays are arranged to be taken whilst the annual cleaning, with its consequent closing of some of the wards, takes place, but this year none of the wards has been closed, and the work has increased instead of lessening. It was therefore necessary to engage several private nurses at two guineas a week. These were difficult to obtain, and on one occasion Miss Byles applied to eleven institutions. Her experience is that trained nurses are practically impossible to obtain, and since the commencement of the war she has always had vacancies for sisters and staff nurses. To fill the vacancies in the trained staff the infirmary committee have now authorised the utilisation of the services of senior probationers and the employment of as many extra probationers as can be accommodated.

There are few of our readers who are bold enough in these busy times for all matrons, and especially for hospital matrons, to suggest that the latter, by good arrangement, could manage to give two to four hours weekly for "patrol work." One matron, however, of a large Poor-Law infirmary in London gives four hours weekly of her off-duty time, and we are told that a number of responsible women are badly needed for this work. The organisation is in the hands of the National Union of Women Workers, Parliament Mansions, Victoria Street, S.W., and we mention the matter in case it may be possible that there are other matrons able and willing to give two hours weekly, if not four, to this service. The police appreciate the patrols so highly that they have asked when on certain beats to have two women patrols associated with them.

The committee of the Imperial Nurses' Club, having now taken premises at 137 Ebury Street, S.W., appeal for £5,000 to defray the initial expenses. It is hoped that the subscriptions of members will render the club self-supporting in time, but the he'p of the public is needed to set it going. The aim of the promoters is to provide a homelike and attractive centre to which all members of the nursing profession, whatever their rank, can come for relaxation and rest of body and mind. The scheme should appeal to all who realise the noble and self-sacrificing work of the best members of the nursing profession. Such a club, rightly conducted, should prove a boon to its members. Miss C. H. Mayers is the honorary secretary.

